# Visio-Spatial Skills in Amateur Taekwondo Athletes Compared with Non-Athletes: A Cross-Sectional Observational Study

**DOI:** 10.3390/vision10030038

**Published:** 2026-06-30

**Authors:** Moeketsi Robert Mohlakoana, Gerrit Jan Breukelman, Lourens Millard

**Affiliations:** Department of Human Movement Science, Faculty of Science, Agriculture and Engineering, University of Zululand, KwaDlangezwa 3886, South Africa; breukelmang@unizulu.ac.za (G.J.B.); millardl@unizulu.ac.za (L.M.)

**Keywords:** taekwondo, visio-spatial skills, accommodation facility, saccadic eye movement, speed of recognition, hand–eye coordination, peripheral awareness, visual memory, sport vision, combat sport

## Abstract

**Background**: Visio-spatial skills (VSS) are fundamental perceptual–cognitive capacities that enable athletes to process dynamic visual information, interpret spatial relationships, and execute precise motor responses under competitive conditions. In taekwondo, where scoring actions are executed within milliseconds and success depends on the rapid detection and anticipation of an opponent’s movements, well-developed VSS are considered a functional prerequisite for performance. **Method**: This cross-sectional observational study examined differences in VSS between amateur taekwondo athletes (*n* = 50) and non-athletes (n = 50) recruited from the King Cetshwayo Municipality District, KwaZulu-Natal, South Africa. Six VSS were assessed using standardized, validated instruments: accommodation facility (AF), saccadic eye movement (SEM), speed of recognition (SR), hand–eye coordination (HEC), peripheral awareness (PA), and visual memory (VM). Between-group comparisons were performed using the Mann–Whitney U test, with effect sizes reported as rank-biserial correlations (r). **Results**: Taekwondo athletes demonstrated significantly superior performance across all VSS domains (all *p* ≤ 0.05), with a large effect observed for SR (r = 0.91), HEC (r = 0,87), SEM (r = 0.78), and AF (r = 0.74), and moderate effect for VM (r = 0.58) and PA (r = 031). The largest between-group percentage differences were observed for SR (79.43%), HEC (48.13%), and SEM (33.33%), with smaller but significant differences in AF (31.05%), VM (14.92%), and PA (4.23%). Pearson correlation analysis revealed a dense, globally integrated VSS network in non-athletes anchored by AF, contrasting with a sparse pattern in taekwondo athletes, in which SEM showed the greatest number of moderate or stronger associations with other variables. These within-group correlation structures are presented as preliminary and descriptive observations only. Intraclass correlation coefficients were excellent for five of the six VSS (ICC ≥ 0.963), with PA yielding a point estimate of ICC = 0.853, characterized by a wide confidence interval. **Conclusions**: These findings indicate that amateur taekwondo athletes show superior perceptual, oculomotor, and visuo-motor performance compared to non-athletes. Within-group VSS correlation pattern differs descriptively between the groups. The cross-sectional design cannot determine whether these differences reflect training-associated perceptual adaptation, pre-existing trait-based self-selection into taekwondo, or a combination of both mechanisms. Both interpretations carry applied implications for talent identification and vision training program design. A longitudinal investigation is required to establish causal directionality. SEM and AF are proposed as the most diagnostically informative VSS markers for taekwondo screening, under either interpretation.

## 1. Introduction

Visual processing underpins the ability of athletes to perceive, interpret, and respond to dynamic sport-specific stimuli. In combat sports, where competitive success depends on the rapid detection and anticipation of an opponent’s movements, the integrity of the visual system is considered a primary determinant of performance [1,2,3]. Visio-spatial skills (VSS) encompass accommodation facility (AF), saccadic eye movement (SEM), speed recognition, hand–eye coordination (HEC), peripheral awareness (PA), and visual memory (VM). Collectively, they enable athletes to orient gaze efficiently, extract predictive kinematic cues, and translate perceptual information into precise motor responses under time-constrained conditions [3,4].

Taekwondo is a World Anti-Doping Agency (WADA)-compliant Olympic combat discipline characterized by rapid, high-amplitude kicking techniques delivered across a broad vertical and spatial attack framework, including the head and trunk of the moving opponent [5,6]. Unlike boxing, which is predominantly restricted to upper-body strikes within a relatively stable interpersonal distance, taekwondo requires athletes to continuously modulate engagement distance through forward surges, backwards retreats, and spinning or high-velocity kick combinations [5,7]. This unique competitive structure places substantial and varied demands on the visual system, including rapid near focus transitions, high-frequency gaze reorientation between lower-limb and trunk cues, instantaneous stimulus discrimination within complex multidirectional movement patterns, and accurate spatial recall of recurring tactical sequences [8,9].

Prior cross-sectional research examining VSS in other sports provides a useful comparative baseline [10]. Studies of amateur boxing have reported accommodation facility (AF) advantages of approximately 18% and speed of recognition (SR) advantages of approximately 88% in athletes relative to non-athletes [3]. Investigations in amateur netball have documented AF advantages of approximately 19%, hand–eye coordination (HEC) advantages of approximately 16%, and peripheral awareness (PA) advantages of approximately 11% [11]. In amateur rugby, saccadic eye movement (SEM) advantages of approximately 19% have been reported [12]. Across these studies, the direction of effects is consistent: sport-trained athletes outperform non-athletes across VSS domains, but the magnitude and profile of differences vary by sport, likely reflecting discipline-specific visual demands. Taekwondo’s unique competitive structure suggests it may place demands on a particularly broad range of VSS systems, a hypothesis this study tests directly. Despite growing interest in sport vision research, comparative studies systematically examining VSS in taekwondo athletes relative to non-athletes remain limited, particularly within the African population [3,10]. The majority of published work has focused on boxing [3], netball [11], and rugby [12], with taekwondo largely under-represented in empirical literature.

However, an important methodological consideration inherent to all cross-sectional athlete versus non-athlete comparisons is the potential confound of self-selection bias. Individuals with superior baseline VSS may be more likely to gravitate toward, persist in, or excel within precision-demanding combat sports such as taekwondo, independently of any training-associated change. Consequently, observed between-group differences may reflect pre-existing perceptual traits that predispose individuals to athletic self-selection rather than, or in addition to, experience-related perceptual differences. Therefore, the present study is explicitly framed as a baseline cross-sectional investigation. This is a methodologically necessary first step for documenting whether systematic VSS differences exist in this understudied population, with the explicit recognition that causal directionality requires prospective longitudinal confirmation. Accordingly, the objectives of this study were (1) to compare VSS performance between amateur taekwondo athletes and non-athletes across six validated assessment domains; (2) to characterize the within-group correlation structure and network topology of VSS in each group; and (3) to determine the intraclass reliability of VSS assessments for discriminating taekwondo athletes from non-athletes. The findings are expected to inform talent identification frameworks, sport-specific vision training program design, and the theoretical understanding of perceptual cognitive differences associated with combat sport participation.

## 2. Materials and Methods

### 2.1. Study Area

King Cetshwayo District Municipality occupies a secondary tier within South Africa’s urban hierarchy. It is classified as a predominantly rural district municipality within KwaZulu-Natal Province, with its largest urban center, Richards Bay [13]. The district context is relevant to study interpretation because it implies a research population that is neither representative of South Africa’s largest metropolitan centers (such as eThekwini/Durban or Johannesburg) nor of entirely rural or high-poverty communities; it occupies an intermediate socio-economic position that may affect access to sport infrastructure and the competitive depth of the taekwondo population sampled.

Taekwondo is a minority sport in South Africa relative to the country’s dominant codes of association football, cricket, and rugby union [14]. It is governed nationally by Taekwondo South Africa, which is affiliated with World Taekwondo and the South African Sports Confederation and Olympic Committee (SASCOC) [15]. Participation has grown incrementally following South Africa’s increasing Olympic engagement with the sport, though practitioner numbers remain substantially lower than in East and Southeast Asian nations where taekwondo is culturally embedded [16]. Within KwaZulu-Natal, taekwondo is practiced primarily in urban and peri-urban clubs, with the King Cetshwayo district representing a typical provincial club environment rather than a high-performance national training center. This context implies that the present sample reflects a recreational-to-amateur competitive population, which should be considered when extrapolating findings to elite or nationally representative athlete groups.

### 2.2. Study Design

The study followed a cross-sectional observational design examining differences in VSS between amateur taekwondo athletes and non-athletes. The study was reviewed and approved by the Institutional Review Board of the University of Zululand (UZ-REC 0691-008 PGD 2025/12) and conducted in accordance with the principles of the Declaration of Helsinki. All participants provided written informed consent prior to participation.

### 2.3. Participants

**Figure 1:**
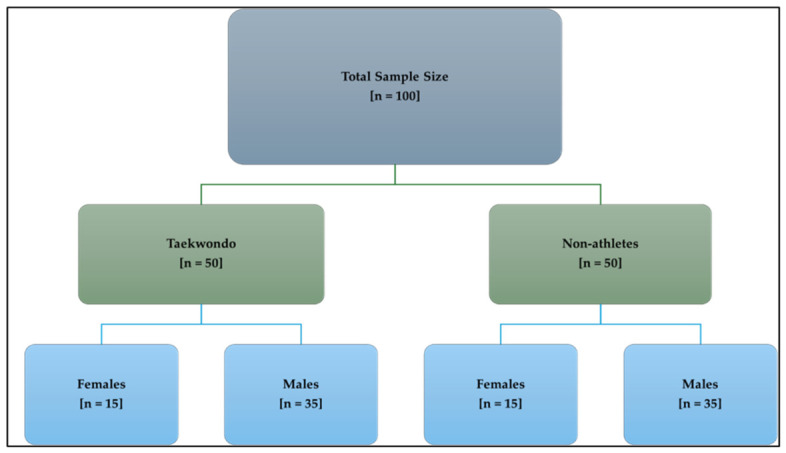
Description of the sample size.

One hundred participants (n = 50 taekwondo and n = 50 non-athletes), aged 18–34 years, were recruited from the King Cetshwayo Municipal District, KwaZulu-Natal, South Africa. Groups were matched for gender distribution (35 males [70%] and 15 females [30%] per group (Figure 1). Taekwondo athletes were required to have a minimum of six months of formal, continuous discipline-specific training. Detailed athlete characterization is provided in Table 1, including belt rank, weekly training volume, and competition experience. Non-athletes had no history of formal sport participation.

Inclusion required normal vision acuity or corrected-to-normal vision (20/20 or better) [17]. Exclusion criteria included a history of neurological disorders, uncorrectable visual impairments, prior exposure to (VSS) testing, and engagement in strenuous physical exercise within 48 h of testing. All participants underwent preliminary optometric screening using Spectrum Eyecare software (version 6.0.0; Digital Optometry, Republic of South Africa) to confirm visual eligibility, followed by a comprehensive ocular examination to verify the structural integrity of the visual system [3].

### 2.4. Procedures

Standard testing procedures were implemented to minimize dietary, physical, and psychological confounders. All assessments were conducted on weekend mornings (07:00–14:00), following a 9–12 h overnight fast [17], in a quiet, well-lit environment under controlled conditions [18]. Test order was standardized across all participants (AF–SEM–SR–HEC–PA–VM). Standardization of test order, rather than randomization, was adopted to ensure procedural consistency and to replicate the sequence used in prior comparable studies [3,11,12]. This approach carries an acknowledged limitation: any systematic fatigue, arousal, or learning effects accumulate in the same direction across all participants, and if athletes and non-athletes differ in their response to task sequencing, for instance, if non-athletes show greater performance degradation with successive tasks, between-group differences on later assessments (PA, VM) may partly reflect sequencing effects rather than genuine VSS differences. Future studies should employ counterbalanced or randomized test orders to mitigate this threat to internal validity.

Each participant completed two trials per test, and the higher score was retained. This approach was adopted to capture each participant’s functional ceiling and minimize the influence of familiarization effects, consistent with prior sports vision research [3,11,12]. However, retention of the best trial rather than the trial mean introduces a potential upward bias in absolute score estimates. Critically, this bias may be differential if athlete and non-athlete groups differ in their between-trial variability or learning rate; if non-athletes show greater improvement from trial 1 to trial 2 (larger familiarization gains), best-trial retention could inflate non-athlete scores disproportionately, thereby underestimating between-group differences, or the reverse if athletes adapt more rapidly. Future studies should adopt mean-trial scoring and report within-participant trial-to-trial variability to enable assessment of this potential confound.

#### 2.4.1. Optometric Assessment

**Figure 2:**
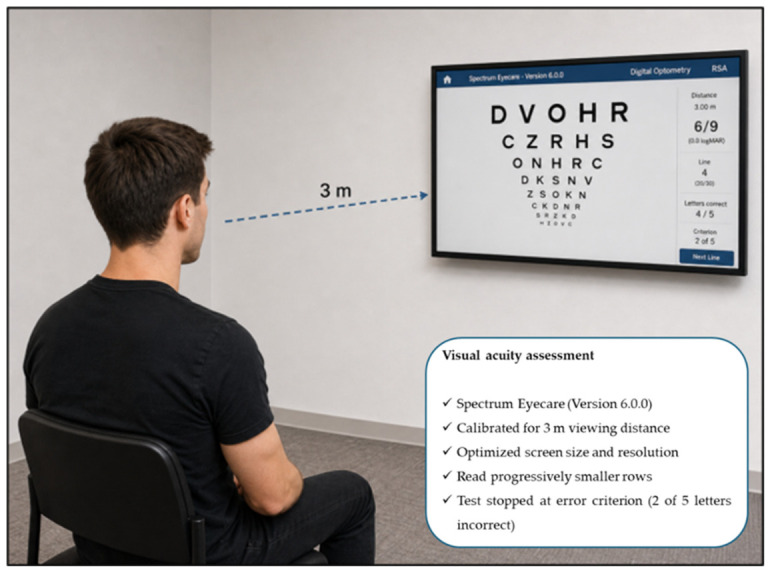
An illustration for visual acuity assessment using Spectrum Eyecare software.

Visual acuity was assessed using Spectrum Eyecare software (Version 6.0.0, Digital Optometry, Port Elizabeth, South Africa), calibrated for 3 m viewing distance, with optimized screen size and resolution (Figure 2). Participants were seated 3 m from the display and read progressively smaller rows of optotypes until an error criterion was reached. All outcomes were reviewed by a qualified optometrist to confirm ≥20/20 vision in all participants [11].

#### 2.4.2. Accommodation Facility (AF)

**Figure 3:**
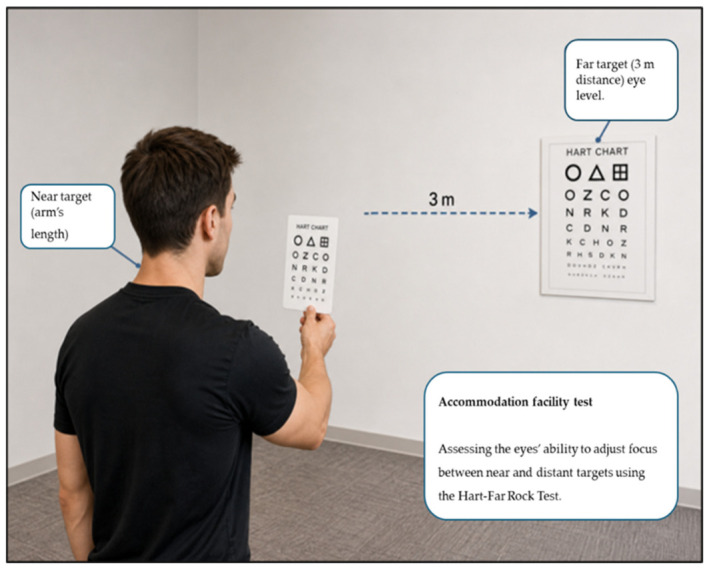
An illustration for accommodation facility assessment using the Hart Near-Far Rock Test.

Accommodation facility, reflecting the eyes’ ability to adjust focus between near and distant targets, was assessed using the Hart Near-Far Rock Test [11] (Figure 3). A large Hart Chart (Bernell Corp., Mishawaka, IN, USA) was positioned on a wall at eye level, 3 m from the participant; a smaller chart was held at arm’s length. Participants alternately read the first letter of each line from the near and far charts for 30 s; errors were recorded and subtracted from the total possible score. The test demonstrates moderate test–retest reliability (r = 0.724) [17,18].

#### 2.4.3. Saccadic Eye Movement (SEM)

Saccadic Eye Movement was assessed using standardized saccadic eye movement charts [19]. Participants stood 3 m from two wall-mounted charts and vertically identified the first letter on the left chart before rapidly shifting gaze to the corresponding letter on the right chart for 30 s, keeping the head stationary. Errors were subtracted from the total possible score [13,16]. The test demonstrates moderate test–retest reliability (r = 0.703) [17].

#### 2.4.4. Speed of Recognition (SR)

**Figure 4:**
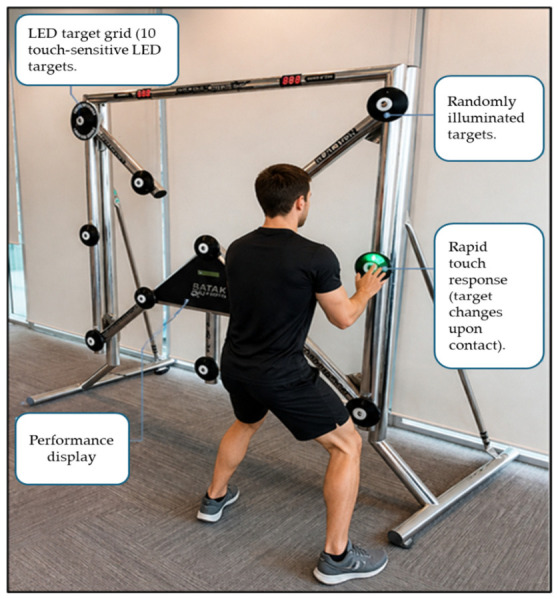
An illustration for speed of recognition assessment using the Evasion function on the Batak Pro system.

Speed of recognition was evaluated using the Evasion function on the Batak Pro system (Quotronics Limited, Horley, Surrey, UK) [17], which has reported reliability of 0.946 [17] (Figure 4). The Batak Pro is a wall-mounted interactive panel of touch-sensitive LED targets arranged in a grid, used to assess visuo-motor performance, including SR, reaction time, HEC, and visual scanning [17]. During assessment, participants respond as rapidly as possible to randomly illuminated LED targets presented at unpredictable spatial locations across central and peripheral fields; each target changed upon contact, prompting an immediate subsequent stimulus. One hundred targets illuminated sequentially over a fixed time interval of 1 s per target [18,19]. The system automatically recorded all errors, applied real-time score deductions, and generated the final score.

#### 2.4.5. Hand–Eye Coordination (HEC)

Hand–eye coordination was evaluated using the Tennis Ball Wall Test, a validated field measure of visuo-motor synchronization and interceptive control [18]. Participants stood 2 m from a wall and executed repeated unilateral throws of a tennis ball, catching the rebound with the contralateral hand, over a 30 s interval, with both hands assessed to determine bilateral accuracy and coordination. Scores ranged from 0 to 60 catches. The test demonstrates moderate reliability (r = 0.708) [17].

#### 2.4.6. Peripheral Awareness (PA)

**Figure 5:**
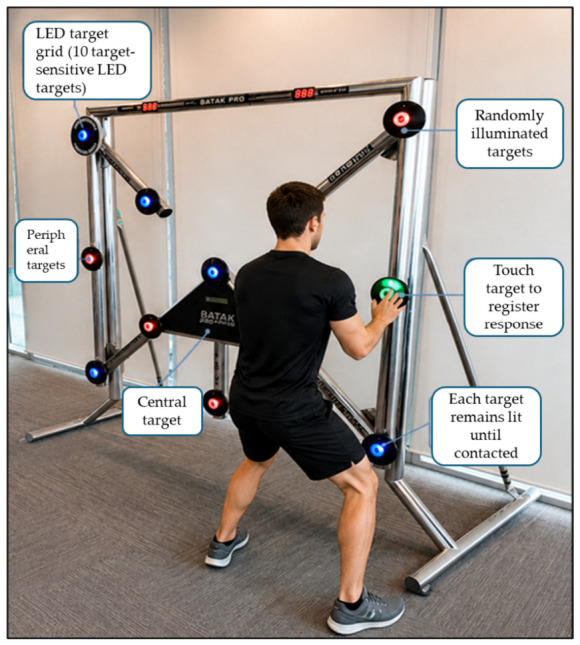
An illustration for peripheral awareness assessment using the Batak Pro system.

Peripheral awareness was assessed using the Batak Pro System Accumulator protocol, in which participants maintained a global visual awareness while rapidly responding to randomly illuminated LED targets presented at central and peripheral locations (Figure 5). Each target remained illuminated until contacted, after which a subsequent target activated at a new spatial position. Two trials were completed with a 5 min recovery interval; the number of correctly executed target contacts within a 60 s period was recorded, and the highest score was retained. The protocol demonstrates high test–retest reliability (r = 0.885) [17].

#### 2.4.7. Visual Memory (VM)

**Figure 6:**
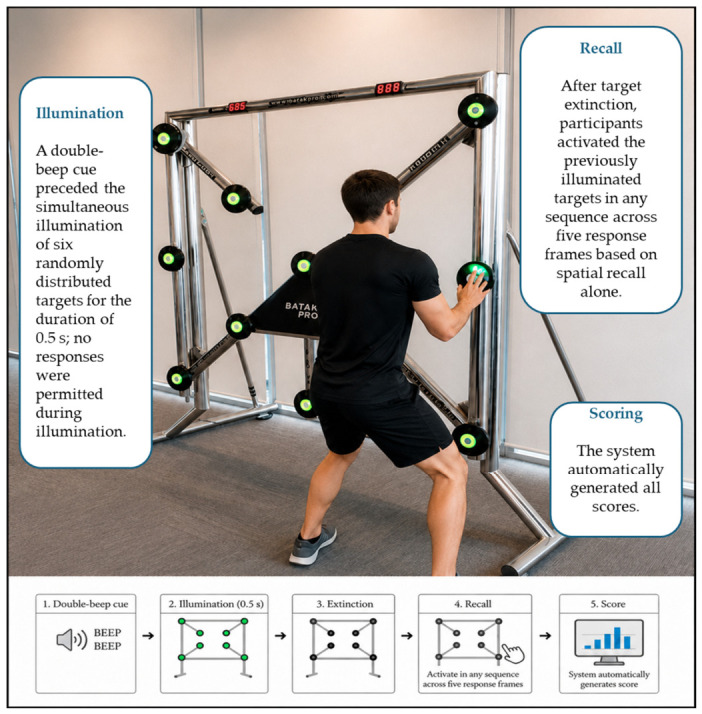
An illustration for visual memory assessment using the Flash Memory protocol on the Batak Pro system.

Visual memory was assessed using the Flash Memory protocol on the Batak Pro system (reliability of 0.735) [17] (Figure 6). A double-beep cue preceded the simultaneous illumination of six randomly distributed targets for 0.5 s; no responses were permitted during illumination. After target extinction, participants activated the previously illuminated targets in any sequence across five response frames based on spatial recall alone. Two trials were performed with a 5 min recovery interval, and the highest score was retained. The system automatically generated all scores.

### 2.5. Statistical Analysis

Quantitative analyses were performed using SPSS version 31.0.1 (IBM Corp., Chicago, IL, USA). Descriptive statistics (mean, standard deviation, range, and percentage difference) were calculated for all variables. Prior to inferential testing, the Shapiro–Wilk test was applied to evaluate the normality assumption for each VSS variable within each group. Because several variables violated the normality assumption, between-group comparisons were performed using the Mann–Whitney U test, which does not require distributional assumptions. Effect sizes for the Mann–Whitney U test were calculated as the rank-biserial correlation (r = 1 − [2U/(n_1_ × n_2_)]). The analysis identified which group demonstrated superior VSS. Pearson correlation analysis was conducted to examine within-group and across-group associations among VSS variables. The resulting within-group matrices are presented as a preliminary observation only. No formal network comparison tests, edge stability analysis, or bootstrap resampling procedures were applied; accordingly, these correlation structures should be treated as hypothesis-generating rather than confirmatory. Statistical significance was set at *p* ≤ 0.05. The intraclass correlation coefficients (ICCs; one-way random model, k = 3 groups), and Cronbach’s α were calculated to determine the reliability of VSS assessment in discriminating between athletes and non-athletes’ groups, classified according to Koo and Li [20] and Nunnally and Bernstein [21].

## 3. Results

### 3.1. Anthropometric Characteristics

Anthropometric data are presented in Table 1. Mean age was comparable across all groups and sexes (range: 20.20 ± 1.61 to 22.93 ± 2.34 years). Body mass was uniform across groups (67–71 kg across both sexes). A progressive increase in stature was observed from non-athletes to taekwondo athletes in both sexes, with non-athlete males recording the lowest mean height (1.69 ± 0.05 m). BMI followed an inverse pattern; non-athletes recorded the highest values (males: 24.06 ± 0.62 kg/m^2^; females: 24.02 ± 0.53 kg/m^2^) and taekwondo athletes the lowest (males: 22.24 ± 0.69 kg/m^2^; females: 22.13 ± 0.49 kg/m^2^), with all values within a healthy range. Non-athletes had no formal training experience. Taekwondo athletes had equivalent training histories (2.13–2.19 years), with belt ranks ranging from 8th Geup (white belt) to 4th Geup (blue belt), a mean weekly training volume of 4.2 ± 1.1 sessions per week, and a mean of 3.4 ± 2.1 competitive bouts over the preceding 12 months.

### 3.2. Visio-Spatial Skills Comparison

Descriptive statistics, between-group comparisons, and effect sizes for VSS are presented in Table 2. Taekwondo athletes demonstrated higher mean scores than the non-athlete group across all six VSS domains. The largest between-group percentage difference was observed for SR (79.43%; *p* = 0.001; r = 0.91), followed by HEC (48.13%; *p* = 0.001; r = 0.87), SEM (33.33%; *p* = 0.001; r = 0.78), AF (31.05%; *p* = 0.001; r = 0.74), VM (14.92%; *p* = 0.001; r = 0.58), and PA (4.23%; *p* = 0.011; r = 0.31). Effect sizes were large for SR, HEC, SEM, AF, and VM, and moderate for PA.

### 3.3. Pearson Correlation Matrix

The Pearson correlation matrix for VSS across both groups is presented in Table 3. In the present study, the suffixes “-N” and “-T” are used throughout to denote non-athlete and taekwondo athlete group membership, respectively (e.g., AF-N = accommodation facility in non-athletes, SEM-T = saccadic eye movement in taekwondo athletes). Within non-athletes, all correlations were positive and directionally consistent, indicating a globally coherent VSS pattern. The strongest within-group association was between AF-N and PA-N (r = 0.52), with AF-N also demonstrating moderate correlations with SEM-N (r = 0.39) and VM-N (r = 0.39). SEM-N showed a moderate cross-group correlation with SEM-T (r = 0.48). The strongest coefficient in the entire matrix was the cross-group AF-N × SEM-T pairing (r = 0.54).

Within taekwondo athletes, the internal correlation structure was markedly sparse, with no within-group pair exceeding r = 0.12. SR-T was largely uncorrelated with all the other taekwondo variables (r = −0.03 to 0.11). A negative cross-group association was observed between PA-T and SEM-T (r = −0.40), and predominantly negative taekwondo to non-athletes cross group correlation’s indicated substantial differences in VSS rank orderings between the groups.

### 3.4. Visio-Spatial Skills Network

**Figure 7:**
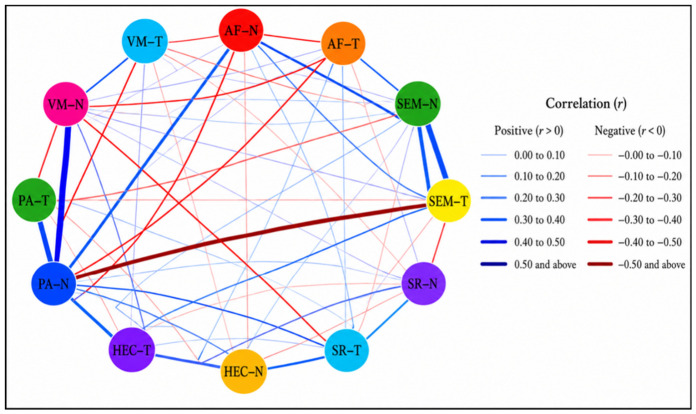
Visio-spatial skills network in taekwondo and non-athletes. Edge color indicates the direction (blue = positive, red = negative); edge thickness indicates the magnitude of correlation (r). AF—accommodation facility; SEM—saccadic eye movement; SR—speed of recognition; HEC—hand–eye coordination; PA—peripheral awareness; VM—visual memory; N—non-athletes; T—taekwondo.

A preliminary correlation pattern analysis examined relationships among visio-spatial skill (VSS) components in taekwondo athletes and non-athletes (Figure 7). The analysis was exploratory and descriptive only, with no formal stability or comparison testing; therefore, findings should be interpreted cautiously. Non-athletes showed a more densely interconnected VSS network, whereas taekwondo athletes displayed fewer but stronger associations, particularly involving saccadic eye movement. The strongest observed relationship was a negative correlation between non-athlete peripheral awareness and athletes’ saccadic eye movement, although cross-group associations may reflect group differences rather than functional relationships. Overall, the findings suggest possible differences in VSS interaction patterns between groups, but larger studies are needed to confirm these preliminary trends.

### 3.5. Reliability

Reliability indices for the taekwondo vs. non-athletes’ comparisons are presented in Table 4. Five of the six VSS demonstrated excellent ICC classifications (ICC ≥ 0.963; α ≥ 0.976) with narrow 95% CIs and small standard errors of measurement (SEM ≤ 1.30 units), confirming that scores on these subscales were highly consistent and reproducible across measurement occasions. PA was the sole exception, yielding an ICC of 0.853 (good classification) with a wide 95% CI (0.266–0.928), and a good α (0.853), consistent with relatively small between-group differences on the subscale (F = 6.79; *p* = 0.011). The wide confidence interval for PA indicates substantial uncertainty around the point estimate and warrants cautious interpretation of the consistency estimate for this skill. Particularly, the comparatively good between-group separation on PA (F = 6.79; *p* = 0.011) relative to all other skills (F ≥ 41.81; *p* < 0.001) reflects limited group differentiation on this skill, as indicated by the F-ratio rather than the ICC, which remains a measure of score consistency rather than discriminative capacity.

### 3.6. Training Duration vs. VSS Scores for Taekwondo

Pearson product-moment correlation analyses revealed no statistically significant associations between training duration and any of the assessed VSS domains (all *p* > 0.05; Table 5). Accommodation facility (AF-T) showed a trivial negative correlation with training (r = −0.036, *p* = 0.805). Similarly, saccadic eye movement (SEM-T) showed a negligible positive association (r = 0.030, *p* = 0.837). Speed of recognition (SR-T) and hand–eye coordination (HEC-T) also demonstrated weak positive correlations with training (r = 0.071, *p* = 0.622; r = 0.058, *p* = 0.690, respectively). Peripheral awareness (PA-T) showed the strongest relationship in the analysis, although the association remained weak and non-significant (r = −0.212, *p* = 0.139). Visual memory (VM-T) demonstrated virtually no relationship with training duration (r = 0.043, *p* = 0.976). The overall VSS composite score showed a near-zero correlation with training exposure (r = 0.004, *p* = 0.976).

## 4. Discussion

This study provides a systematic cross-sectional characterization of VSS and their correlation structure in amateur taekwondo athletes and non-athletes. The principal findings are fourfold. First, taekwondo athletes demonstrated significantly superior VSS across all six domains, with large effects for SR (r = 0.91), HEC (r = 0.87), SEM (r = 0.78), and AF (r = 0.74), a moderate effect for VM (r = 0.58), and a smaller effect for PA (r = 0.31). Second, within-group VSS correlation patterns differed between groups: non-athletes showed a dense, predominantly positive structure anchored by AF, whereas taekwondo athletes showed a sparser configuration in which SEM demonstrated the greatest number of moderate associations; these observations are descriptive and exploratory. Third, no significant association was found between training duration and VSS outcomes, although this finding is constrained by a restricted range and limited statistical power. Fourth, reliability was excellent for five VSS components, while PA showed wider confidence intervals and should be interpreted cautiously.

A key interpretive constraint is that the observed between-group differences can be explained by two non-mutually exclusive mechanisms. The training-association account proposes that repeated exposure to sport-specific perceptual demands may reorganize VSS functioning toward task-relevant configurations [3,11,12] (Table 6). In contrast, the self-selection account suggests that individuals with more efficient baseline perceptual profiles may be more likely to enter and persist in taekwondo due to early performance advantages [24,25]. These competing interpretations cannot be resolved without prospective longitudinal data. Both have applied relevance and are not mutually exclusive, as they may operate concurrently within the same population. Therefore, the cross-sectional design limits the ability to adjudicate between these explanations.

The absence of a significant training duration between VSS relationships is relevant to this interpretation. While a dose–response framework would predict a positive association between accumulated practice and performance [22], no such trend was observed. However, this result is limited by restricted variability in training duration (approximately 2 years) and low statistical power (post hoc power < 0.30 for r ≈ 0.15). Moreover, within a pre-selected athletic sample, the absence of association does not exclude early-stage adaptation effects or pre-existing differences prior to sport entry. Longitudinal designs spanning novice to elite levels are required to clarify developmental trajectories.

Both interpretations have applied relevance. If VSS differences are training-sensitive, targeted vision training may enhance SR, HEC, and SEM. If they reflect pre-existing traits, VSS profiling may support talent identification, particularly using AF and SEM as potential discriminators.

### 4.1. Anthropometric Profile

Sex distribution was comparable across groups (70% male, 30% female), reducing sex-related confounding [7]. Age (20.20–22.93 years) and body mass (67–71 kg) were also similar, controlling for known age- and mass-related effects on visuo-motor performance [22,23]. A trend of higher stature in taekwondo athletes aligns with combat-sport morphological profiles [26], while lower BMI in athletes compared to non-athletes is consistent with inverse associations between BMI and visuo-motor speed [7,22]. Training exposure was equivalent across athlete groups (2.13–2.19 years), supporting comparability.

### 4.2. Accommodation Facility (AF)

Taekwondo athletes demonstrated a 31.05% higher AF relative to non-athletes (39.74 ± 2.68 vs. 27.40 ± 3.89; *p* = 0.001; r = 0.74). This magnitude exceeds the 18% difference previously reported in amateur boxing [3] and the 19% difference in amateur netball [11,27] (Table 6), suggesting that taekwondo participants show comparatively is associated with comparatively greater between-group AF differences. The sport’s continual modulation of engagement distance via forward surges, backward retreats, and long-range kicking exchanges places repeated demands on near-far focus alternation, which may be relevant to AF performance [28,29,30]. These AF differences are consistent with both interpretive frameworks; repeated near-far focus cycling associated with taekwondo competition may be related to accommodative performance differences [31,32], and/or individuals with faster accommodative responses may experience early competitive success in taekwondo more readily and therefore persist in the sport [25]. The cross-sectional design does not permit these possibilities to be distinguished.

### 4.3. Saccadic Eye Movement (SEM)

Taekwondo athletes demonstrated a 33.33% difference in SEM relative to non-athletes (48.06 ± 6.08 vs. 32.04 ± 5.01; *p* = 0.001; r = 0.78), marginally exceeding the 32.0% difference reported in amateur rugby [12]. The sport demands rapid gaze shifts between the opponent’s trunk, hips, stance base, and attacking limb to detect kinematic cues such as hip rotation or weight transfer [8,33]. Research on micro-saccadic function further suggests a role for fine oculomotor control in pre-motor attentional modulation [34]. The descriptive observation that SEM showed the greatest number of moderate associations within the taekwondo group correlation is consistent with the possibility that saccadic efficiency is particularly relevant to taekwondo participation [35]. Whether this reflects experience-associated differences, a pre-existing oculomotor trait that facilitates sport entry and retention, or both, cannot be determined from the present data [25,35].

### 4.4. Speed of Recognition (SR)

The 79.43% difference in SR (52.42 ± 6.76 vs. 10.78 ± 5.50; *p* = 0.001; r = 0.91) represents the largest absolute between-group difference observed. Although marginally lower than the 88% difference reported in amateur boxing [3], the magnitude in taekwondo is substantial and broadly consistent with prior observational literature [36]. Athletes who regularly practice in high-speed visual environments may develop more efficient perceptual discrimination and faster visio-spatial judgment [8]. Alternatively, individuals with faster perceptual processing may self-select into sports where such capacities are advantageous [25]. The present design cannot establish which account better explains the observed association.

### 4.5. Hand–Eye Coordination (HEC)

The 48.13% higher HEC in taekwondo athletes (37.36 ± 4.63 vs. 19.38 ± 4.06; *p* = 0.001; r = 0.87) distinctly exceeds the 16% difference reported in amateur netball [11], consistent with the view that combat sports place particularly high demands on rapid visuo-motor coupling. Unlike netball, where ball trajectories arise from structured team patterns, taekwondo requires continuous motor recalibration in response to unpredictable opponent cues [8,36]. Observational and experimental evidence suggests shorter reaction times and enhanced anticipatory skill in karate athletes [37] and faster visual search efficiency in taekwondo athletes [36,38], though the mechanisms underlying these differences remain to be established. The directionality of the HEC association observed here likewise cannot be determined from the present cross-sectional data.

### 4.6. Peripheral Awareness (PA)

Taekwondo athletes demonstrated a good but statistically significant 4.23% higher PA score (57.20 ± 5.07 vs. 54.78 ± 4.17; *p* = 0.011; r = 0.31). This is the smallest between-group difference observed and should be interpreted cautiously, given the wide ICC confidence interval (0.266–0.928) and the moderate effect size. The relatively small PA difference is consistent with the paired, forward-oriented structure of taekwondo competition, in which the opponent remains predominantly within the central visual field rather than at the lateral periphery [39,40]. Athletes appear to prioritize fixation on the trunk and hip region to extract predictive kinematic cues, which may reduce the functional need for broad peripheral expansion relative to multi-player invasion sports [33]. Given the limited between-group discrimination and the reliability concerns observed for PA, this variable should be interpreted cautiously both as a research outcome and as a potential screening marker in taekwondo populations.

### 4.7. Visual Memory (VM)

Taekwondo athletes demonstrated a higher VM score (52.16 ± 6.64 vs. 44.38 ± 5.32; *p* = 0.001; r = 0.58), exceeding the non-significant differences reported in boxing [3], netball [11], and rugby [12] (Table 6). The dyadic, tactically repetitive structure of taekwondo competition, in which movement patterns, stance shifts, and feint-attack sequences recur across bouts, may be relevant to the observed VM differences [41]. Research on sport-specific cognitive processes has found enhanced information retention under consistent combat constraints [42,43]. Whether the VM difference observed here reflects experience-related differences in spatial recall, pre-existing individual differences in visual memory, or both cannot be established from the present design.

### 4.8. VSS Network Organization

Correlation patterns differed descriptively between groups. Non-athletes showed a more densely interconnected structure anchored by AF, whereas taekwondo athletes showed a sparser configuration with SEM as the most connected variable. These findings are exploratory; no network stability or inferential procedures were applied [44,45]. The negative SEM-T–PA-T association in athletes (r = −0.40) may reflect divergent perceptual strategies, such as peripheral-dominant versus foveal-focused scanning, although this remains speculative without eye-tracking evidence [46,47]. These patterns may reflect either training-related functional reorganization or self-selection based on pre-existing perceptual traits [46,48]. Longitudinal work with gaze-based measures is required to distinguish these mechanisms [48,49].

### 4.9. Training Duration vs. VSS Scores

As documented in Section 3.6, no statistically significant dose–response relationship was observed between training duration and any individual VSS component or the composite VSS score. This null finding must be interpreted with caution rather than as positive evidence for either competing explanation. Under a dose–response framework [49], one would anticipate a positive monotonic relationship between accumulated practice and performance, yet no such gradient was detected here.

However, the restricted variance in training duration (approximately 2 years across both sexes), combined with inadequate statistical power to detect small correlations (post hoc power < 0.30 for r~0.15 at α = 0.05), substantially limits the inferential value of this analysis. Furthermore, the absence of a dose–response relationship within a sample that has already undergone sport selection (i.e., individuals who have persisted in taekwondo for at least six months) does not rule out the possibility that VSS differences emerge during early exposure or prior to formal sport entry. Recruiting participants across a wider training continuum from novices (<6 months) to advanced athletes (>5 years) and measuring VSS longitudinally would provide substantially greater leverage for evaluating this question.

### 4.10. Limitations

This study has several limitations. First, and most critically, the cross-sectional design cannot resolve whether the observed VSS superiority in taekwondo athletes reflects training-associated differences, pre-training trait-based self-selection into taekwondo, or their interaction. Three study designs would provide stronger evidence regarding directionality: (a) a prospective longitudinal study measuring VSS in taekwondo novices before and after a structured training period; (b) a randomized controlled trial assigning participants to taekwondo training versus an active control condition; or (c) a prospective cohort study tracking VSS in individuals prior to sport specialization, comparing those who subsequently select taekwondo against those who do not. Until such data are available, the present findings are best interpreted as establishing that systematic VSS differences are associated with taekwondo participation, with causal directional remaining an open question.

Second, the sample was drawn from a single municipal district in South Africa, which may limit generalizability to other populations, competitive levels, and cultural contexts. Third, gender-stratified analyses were not conducted due to sample size constraints; future studies should be adequately powered to examine sex-specific VSS profiles. Fourth, the absence of competitive-level stratification means that dose–response relationships between training intensity and VSS differences cannot be characterized. Fifth, the retention of the best of two trials rather than the trial mean may inflate absolute performance estimates and should be reconsidered in future work. Sixth, the test order was standardized rather than randomized, which may introduce systematic learning or fatigue effects across the assessment sequence. Seventh, no eye-tracking or gaze behavior data were collected; such data would provide direct evidence relevant to the correlation patterns observed between VSS components, particularly the PA-T × SEM-T association. Eighth, the exploratory correlation analysis was not supported by formal statistical comparison procedures, and the correlation structures should be treated as preliminary descriptions requiring replication with larger samples and appropriate stability-testing methods.

## 5. Conclusions

This study demonstrates that amateur taekwondo athletes show significantly superior VSS performance compared with non-athletes across all six assessed domains, with large effects for SR, HEC, SEM, AF, and VM, and a moderate effect for PA. Beyond individual skill magnitudes, within-group VSS co-variation patterns differ descriptively between groups from the globally coherent, AF-anchored pattern observed in non-athletes to a sparser, SEM-associated pattern in taekwondo athletes, though these observations are preliminary and exploratory only.

These differences are consistent with either training-associated perceptual adaptation or pre-training trait-based self-selection into taekwondo, or a combination of both. The cross-sectional design cannot adjudicate between these competing explanations. The absence of a significant training duration-VSS relationship within the present sample further highlights the need for caution in attributing the observed differences to training exposure alone, though the null result is inconclusive given the restricted range and low statistical power of that analysis.

Both interpretations carry applied value. If VSS differences are associated with sport participation, they motivate structured sport vision training programs targeting SR, HEC, and SEM. If they reflect self-selection, they validate VSS screening, particularly AF and SEM, identified here as the most diagnostically informative markers as talent identification instruments applicable at athlete entry points before meaningful training has occurred. The negative within-athlete PA-SEM correlation provides a preliminary and practically relevant observation for vision training program design, suggesting that simultaneously optimizing broad peripheral monitoring and rapid targeted saccadic efficiency may require explicitly differentiated training strategies.

Reliability was Excellent for five of six VSS; PA alone warrants cautious interpretation due to its good between-group discrimination and wide ICC confidence interval. Future prospective studies incorporating eye-tracking assessments, gender-stratified designs, formally stability-tested correlation analyses, and wider training duration ranges are recommended to elucidate the mechanisms, directionality, and dose–response characteristics of VSS differences in taekwondo and other combat sport disciplines.

## Figures and Tables

**Table 1:** Anthropometric characteristics of taekwondo and non-athletes.

Parameter	Non-Athletes (n = 50)		Taekwondo Athletes (n = 50)	
	Male	Female	Male	Female
Gender, n (%)	35 (70%)	15 (30%)	35 (70%)	15 (30%)
Age (y)	21.65 ± 2.43	22.93 ± 2.34	22.08 ± 2.39	20.2 ± 1.61
Body Mass (kg)	69.52 ± 5.08	71.10 ± 5.15	68.06 ± 4.33	70.21 ± 3.84
Height (m)	1.69 ± 0.05	1.71 ± 0.05	1.75 ± 0.04	1.78 ± 0.48
BMI (kg/m^2^)	24.06 ± 0.62	24.02 ± 0.53	22.24 ± 0.69	22.13 ± 0.49
Experience (y)	0.00 ± 0.00	0.00 ± 0.00	2.17 ± 0.66	2.13 ± 0.51
Belt Rank (Geup range)			4th–8th	4th–8th
Weekly Training (sessions/wk)	-	-	4.2 ± 1.1	4.2 ± 1.1
Competition Bouts (12 months)	-	-	3.4 ± 2.1	3.4 ± 2.1

BMI, Body Mass Index; y, years; kg, kilogram; m, meters; n, number of participants; ±, standard deviation; %, percent; kg/m^2^, kilograms per square meter.

**Table 2:** Visio-spatial skills comparison between taekwondo and non-athletes.

Visio-Spatial Skills	Non-Athletes (*n* = 50)	Taekwondo (*n* = 50)	Difference (%)	Significance (*p*-Value)	Effect Size (r)	Effect Classification
Accommodation Facility	27.40 ± 3.89	39.74 ± 2.68	31.05	0.001	0.74	Large
Saccadic Eye Movement	32.04 ± 5.01	48.06 ± 6.08	33.33	0.001	0.78	Large
Speed of Recognition	10.78 ± 5.50	52.42 ± 6.76	79.43	0.001	0.91	Large
Hand–Eye Coordination	19.38 ± 4.06	37.36 ± 4.63	48.13	0.001	0.87	Large
Peripheral Awareness	54.78 ± 4.17	57.20 ± 5.07	4.23	0.011	0.31	Moderate
Visual Memory	44.38 ± 5.32	52.16 ± 6.64	14.92	0.001	0.58	Large

Data are presented as mean ± SD. *p*-values derived from Mann–Whitney U test; effect size r = rank-biserial correlation: small ≥ 0.10, moderate ≥ 0.30, large ≥ 0.50.

**Table 3:** Pearson correlation matrix of visio-spatial skills in taekwondo athletes and non-athletes.

	AF-N	AF-T	SEM-N	SEM-T	SR-N	SR-T	HEC-N	HEC-T	PA-N	PA-T	VM-N	VM-T
**AF-N**	1.00											
**AF-T**	−0.16	1.00										
**SEM-N**	0.39	−0.16	1.00									
**SEM-T**	0.54	0.00	0.48	1.00								
**SR-N**	0.18	−0.12	0.15	0.17	1.00							
**SR-T**	−0.24	−0.03	0.12	0.03	−0.07	1.00						
**HEC-N**	0.33	0.17	−0.10	0.18	0.11	−0.05	1.00					
**HEC-T**	−0.19	0.03	−0.18	−0.03	−0.04	0.09	0.02	1.00				
**PA-N**	0.52	−0.25	0.25	0.43	0.15	−0.09	0.31	−0.05	1.00			
**PA-T**	−0.23	−0.04	−0.03	−0.40	−0.08	0.11	−0.19	−0.13	−0.22	1.00		
**VM-N**	0.39	−0.19	0.21	0.28	0.17	−0.07	0.28	0.07	0.31	−0.20	1.00	
**VM-T**	−0.10	−0.12	0.08	−0.13	0.03	0.10	−0.16	0.09	0.06	0.12	0.18	1.00

AF—accommodation facility; SEM—saccadic eye movement; SR—speed of recognition; HEC—hand–eye coordination; PA—peripheral awareness; VM—visual memory; N—non-athletes; T—taekwondo.

**Table 4:** Reliability indices for taekwondo athletes vs. non-athletes across visio-spatial skills assessments.

Combat Sport	VSS	MS Between	MS Within	F-Ratio	ICC	ICC 95% CI	SEM	A	ICC Classification	α Classification
Taekwondo (vs) Non-Athletes	AF	3806.9	11.2	341.20	0.990	0.977–0.999	0.26	0.997	Excellent	Excellent
	SEM	6416.0	31.0	206.75	0.998	0.963–0.998	0.55	0.995	Excellent	Excellent
	SR	43,347.2	38.0	1141.50	0.995	0.993–1.000	0.26	0.999	Excellent	Excellent
	HEC	8082.0	19.0	426.26	0.966	0.982–0.999	0.30	0.998	Excellent	Excellent
	PA	146.4	21.5	6.79	0.853	0.266–0.928	2.35	0.853	Good	Good
	VM	1513.2	36.2	41.81	0.963	0.828–0.988	1.30	0.976	Excellent	Excellent

ICC = intraclass correlation coefficient (one-way random model, k = 3 groups; CI = 95% Confidence interval; α = Cronbach’s Alpha. Classifications: ICC—excellent ≥ 0.90, good 0.75–0.89, moderate 0.50–0.74, poor < 0.50 [22]; α—excellent ≥ 0.90, good 0.80–0.89, acceptable 0.70–0.79 [23]. All between-group F-ratios *p* < 0.001 except PA (*p* = 0.011).

**Table 5:** Training vs. VSS scores for taekwondo and non-athletes.

Visio-Spatial Skills	R	*p*-Value	Significance
AF-T	−0.036	0.805	ns
SEM-T	0.030	0.837	ns
SR-T	0.071	0.622	ns
HEC-T	0.058	0.690	ns
PA-T	−0.212	0.139	ns
VM-T	0.043	0.976	ns
VSS Composite	0.004	0.976	ns

AF, accommodation facility; SEM, saccadic eye movement; SR, speed of recognition; HEC, hand–eye coordination; PA, peripheral awareness; VM, visual memory; VSS, VISIO-SPATIAL SKILL; T, taekwondo athletes; ns, not significant.

**Table 6:** Percentage differences between non-athletes and athletes across visio-spatial skills and sports.

Visio-Spatial Skills	Taekwondo Current Study	Boxing [3]	Netball [11]	Rugby [12]
Accommodation Facility	31.05%	18%	19%	10%
Saccadic eye movement	33.33%	43%	26%	32%
Speed of Recognition	79.43%	88%	55%	93%
Hand–Eye Coordination	48.13%	12%	16%	24%
Peripheral Awareness	4.23%	15%	11%	15%
Visual Memory	14.92%	4%	3%	0.4%

## Data Availability

The data presented in this study are available upon request from the corresponding author to protect the confidentiality of the participants.

## Abbreviations

The following abbreviations are used in this manuscript:

ICC	Intraclass Correlation Coefficient
AF	Accommodation Facility
CI	95% Confidence Interval
HEC	Hand–Eye Coordination
N	Non-athletes
PA	Peripheral Awareness
SEM	Saccadic Eye Movement
SR	Speed of Recognition
T	Taekwondo
VM	Visual Memory
α	Cronbach’s Alpha
VSS	Visio-Spatial Skill
